# Curcumin-loaded chitosan–bovine serum albumin nanoparticles potentially enhanced Aβ 42 phagocytosis and modulated macrophage polarization in Alzheimer’s disease

**DOI:** 10.1186/s11671-018-2759-z

**Published:** 2018-10-22

**Authors:** Rui Yang, Yan Zheng, Qingjun Wang, Liang Zhao

**Affiliations:** 1 The First Affiliated Hospital of Jinzhou Medical University, Jinzhou, 121000 People’s Republic of China; 2 School of Pharmacy, Jinzhou Medical University, Jinzhou, 121000 People’s Republic of China

**Keywords:** Alzheimer’s disease, Blood–brain barrier, Aβ peptide, Nanoparticles, Curcumin

## Abstract

Alzheimer’s disease (AD) is the most common neurodegenerative disorder in the elderly population. In the treatment of AD, some obstacles, including drug penetration difficulty through the blood–brain barrier (BBB), inadequate clearance of the Aβ peptide, and the massive release of inflammatory factors, must be urgently overcome. To solve these problems, we developed special and novel nanoparticles (NPs) made of chitosan (CS) and bovine serum albumin (BSA) to enhance the penetration of drugs through the BBB. Curcumin as a potent anti-inflammatory agent was used to increase the phagocytosis of the Aβ peptide. The results demonstrated that curcumin-loaded CS-BSA NPs effectively increased drug penetration through the BBB, promoted the activation of microglia, and further accelerated the phagocytosis of the Aβ peptide. Furthermore, curcumin-loaded CS-BSA NPs inhibited the TLR4-MAPK/NF-κB signaling pathway and further downregulated M1 macrophage polarization. This study suggested that curcumin-loaded CS-BSA NPs hold the potential to enhance Aβ 42 phagocytosis through modulating macrophage polarization in AD.

## Introduction

Alzheimer’s disease (AD) is a neurodegenerative disorder marked by an insidious onset and a progression in cognitive decline. Histopathologically, amyloid-β (Aβ) 42 as a peptide containing 42 amino acid is aggregated into amyloid β-peptide filbrils characterized by extracellular “senile plaques” in AD, thus inducing neuronal apoptosis and the loss of synapses [1–3]. Generally, Aβ 42 monomers are physiologically soluble and non-toxic, while its oligomers are more toxic in vitro and in vivo [4, 5]. Therefore, intervening Aβ 42 aggregation by clearing the Aβ 42 monomer is widely considered as the most appropriate therapeutic target for AD [6–9]. It is well known that Aβ peptides can trigger microglial activation by interacting with several Toll-like receptors (TLRs), including TLR4, and they also promote CD14-, TLR4-, or TLR2-dependent phagocytosis and the clearance of Aβ 42 [10–12]. Although microglial activation can promote the clearance of Aβ 42, microglial cells—a type of mononuclear macrophage—are possibly over-activated and polarized to the M1 phenotype (characterized by the release of potentially neurotoxic soluble factors and pro-inflammatory cytokines), thus leading to neuronal death and exacerbating the development of AD. Conversely, some microglial cells are of the classically activated (M2) phenotype characterized by the production of anti-inflammatory cytokines; these ameliorate cognitive dysfunction in AD [13–16]. Therefore, the ratio of M1/M2-type macrophages can significantly affect the progression of AD [17, 18]; furthermore, the upregulation required to convert proinflammatory M1 to anti-inflammatory M2 macrophages will show promising potential in the prevention and treatment of AD.

Curcumin, which originates from a component of the Indian spice turmeric (Curcumin longa)—a type of ginger—is a potent anti-inflammatory agent that can reduce inflammation and may even play a role in the treatment of AD [19]. In recent years, it was found that curcumin reportedly possesses anti-amyloidogenic, anti-inflammatory, anti-oxidative, and metal-chelating properties that may have potential neuroprotective effects [20, 21]. Curcumin modulates macrophage polarization through the inhibition of the toll-like receptor 4-mitogen-activated protein kinase (TLR4-MAPK)/NF-κB pathways [22–24]. However, the poor stability and bioavailability of curcumin limit its clinical application. In addition, the presence of the blood–brain barrier (BBB) also prevents the penetration of curcumin in the treatment of AD [25–27].

To enhance drug transportation from the blood to the brain, nanoparticles (NPs) with surface functionalized with peptides [28] and antibodies [29] assists drug delivery across the BBB and the BBB penetration efficiency of NPs could be significantly enhanced via active transport mechanisms other than simple passive diffusion [30]. Chitosan (CS) NPs—a nano-carrier for associating with Aβ—can permeate the BBB and is non-immunogenic [31]. In addition, serum albumin was found in the circulating plasma of the human body at concentrations of 50 g/L of serum, and it was non-toxic and well tolerated by the immune system [32, 33]. It was also reported that bovine serum albumin (BSA)-derived nanoparticles have sustained release properties which can increase the half-life of drug, thereby decreasing the frequency of administration and increasing patient compliance [34]. Therefore, we used CS and BSA as the two biomaterials to prepare curcumin-loaded CS-BSA NPs to achieve the best BBB penetration. The effects of curcumin on the phagocytosis of Aβ 42, inflammatory cytokine secretion, and the regulation of the TLR4-MAPK/ NF-κB pathways were investigated to further confirm the molecular mechanism of curcumin on macrophage polarization.

### Materials

CS with a deacetylation degree of 80% and a molecular weight of approximately 400 kDa was purchased from Haixin Biological Product Co., Ltd. (Ningbo, People’s Republic of China). BSA was purchased from Sigma-Aldrich Co. (St. Louis, MO, USA), and curcumin was purchased from Dalian Meilun Biotechnology Co., Ltd. (Dalian, People’s Republic of China). FITC-β-amyloid (1–42) was purchased from Chinese Peptide Co., Ltd. (Hangzhou, People’s Republic of China). The other purchased chemicals were of analytical grade and obtained from Sigma-Aldrich Co. The macrophage cell line, RAW 264.7 (mouse leukemic monocyte macrophage cell line), and the brain microvascular endothelial cell line (hCMEC/D3), which served as a model of the human BBB, were established by the Shanghai Institute of Cell Biology, Chinese Academy of Sciences, Shanghai (People’s Republic of China). Both cells were maintained at temperatures of 37 °C, and with a 5% CO_2_ atmosphere, in Dulbecco’s Modified Eagle’s Medium (DMEM) supplemented with 10% (volume/volume) heat-inactivated fetal bovine serum and antibiotics (100 U/mL of penicillin and 100 mg/mL of streptomycin). It was reported that RAW 264.7 cells treated with lipopolysaccharide (LPS) recapitulate aspects of microglial cells observed in neurodegenerative diseases exemplified by AD [35, 36]. Therefore, macrophage cell line RAW 264.7 cells polarized to M1 phenotype by lipopolysaccharide (LPS; 1 μg/ml) were further applied to simulate microglial cells in AD.

### Preparation of curcumin-loaded CS-BSA NPs

According to our previous report [37], under an electrostatic interaction, positively charged CS could conjugate with negatively charged BSA to form NPs. The preparation method was as follows: 0.1% acetic acid was used to dissolve CS to obtain a solution at 0.5 mg/mL of CS and 100 μL of DMSO containing 0.05 mg/mL of curcumin was added into the CS solution for thorough mixing under magnetic stirring at room temperature. As an appropriate amount of BSA solution, 1.0 mg/mL was slowly dropped into the mixture of CS and curcumin; at this point, the opalescence phenomenon appeared and CS-BSA NPs were further condensed into solid particles. Moreover, the size, polydispersity, zeta potential, and morphology of the NPs were investigated. In vitro drug release from NPs was estimated using a previously reported method [37]. The encapsulation efficiency (EE, %) of curcumin in NPs was calculated using the equation below.

$$ \mathrm{EE}\%=\frac{W_{\mathrm{total}}-{W}_{\mathrm{free}}}{W_{\mathrm{total}}}\times 100\% $$

*W*_total_was the amount of initially added curcumin, *W*_free_was the amount of curcumin remained in the supernatant.

### Cell apoptosis evaluation by MTT

To determine the safety of CS-BSA NPs on cell apoptosis, an MTT assay was used to evaluate cell viability. According to the protocol of our previous study, different amounts of blank CS-BSA NPs were used to treat RAW 264.7 cells (M1 phenotype) and hCMEC/D3 cells for 24 h at 37 °C for further analysis.

### Penetration studies using an in vitro BBB model

A monolayer transwell culture using the brain microvascular endothelial cell line, hCMEC/D3, is a common in vitro BBB model that is used to study the brain delivery of NPs. The hCMEC/D3 cell mix (in 0.5–1.0 mL of total volume) was added into the insert in the upper chamber of a 12-well transwell plate for monolayer cell culture with a transendothelial electrical resistance > 300 Ω; PBS at a pH level of 7.4 was added into the lower chamber. Free curcumin and the suspension of curcumin-loaded CS-BSA NPs were placed in the upper chamber for continuous incubation for 3 h in an incubator set at 37 °C, 5% CO_2_. Afterward, free curcumin and curcumin-loaded CS-BSA NPs were transferred across the cells and entered in the lower chamber. The quantification of the penetrated NPs was detected using a microplate reader (Synergy-2; BioTek Instruments, Winooski, VT, USA) by checking the fluorescence intensity of curcumin, which is excited at 425 nm and emitted at 530 nm. The relative fluorescence ratio (RFR, %), which represents the penetration rates of NPs, was calculated by determining the ratio of the fluorescence intensity of the penetrated curcumin-loaded CS-BSA NPs in the lower chamber to that of the initially added curcumin-loaded CS-BSA NPs in the upper chamber. Different endocytic inhibitors, such as chlorpromazine (which inhibits clathrin-mediated uptake) at 10 μg/mL, genistein (caveolae-mediated uptake) at 1 μg/mL, cytochalasin D (30 μM, macropinocytosis), and 20 μg/mL of sodium azide (an energy inhibitor), were used to ascertain various endocytic pathways involved in the various penetration mechanisms. The relative penetration ratio was determined by comparing the penetrated rates of NPs treated with inhibitors with those of NPs treated with non-inhibitors.

### The cellular uptake of curcumin-loaded CS-BSA NPs

The distribution and location of curcumin-loaded CS-BSA NPs in RAW 264.7 cells (M1 phenotype) was observed using confocal laser scanning microscopy (FluoView FV10i; Olympus Corporation, Tokyo, Japan). The hCMEC/D3 cell mix (in 0.5–1.0 mL of total volume) was added into the insert in the upper chamber of the 12-well Transwell plate for monolayer cell culture with a transendothelial electrical resistance > 300 Ω. RAW 264.7 cells (M1 phenotype) were seeded in the lower chamber. Free curcumin and a suspension of curcumin-loaded CS-BSA NPs was placed into the upper chamber for continuous incubation in an incubator set at 37 °C, 5% CO_2_. At predetermined intervals, the cellular distributions of free curcumin and curcumin-loaded CS-BSA NPs in RAW 264.7 cells (M1 phenotype) were observed by detecting the green fluorescence emitted by curcumin using confocal laser scanning microscopy.

### Detection of the phagocytosis of Aβ 42 induced by free curcumin and curcumin-loaded CS-BSA NPs

RAW 264.7 cells (M1 phenotype) in full growth media were seeded in a 12-well plate (1 × 10^5^ cells/well) and treated with free curcumin and curcumin-loaded CS-BSA NPs for 24 h at 37 °C. To remove uninternalized curcumin and curcumin-loaded CS-BSA NPs, distilled water was used to wash cells in a short time two times. It was found that curcumin and curcumin-loaded CS-BSA NPs were completely eliminated from the medium, and there was no obvious risk of cells from distilled water induced low osmolarity because the morphology of cells washed by distilled water was intact and no burst of cells was observed. Finally, free FITC-labeled Aβ 42 dissolved in PBS (pH 7.4) was added into the plate for continuous incubation for 3 h. The phagocytosis of FITC-labeled Aβ 42 into RAW 264.7 cells (M1 phenotype) was represented by detecting the green fluorescence emitted by FITC. The intracellular phagocytosis and location of Aβ 42 within the cells were further studied using confocal laser scanning microscopy (FluoView FV10i; Olympus Corporation).

### Western blot assay

To explore the possible molecular mechanism of curcumin-mediated macrophage polarization, we examined the expression levels of pro-inflammatory cytokines, such as tumor necrosis factor (TNF)-α and interleukin (IL)-6, and the phosphorylation levels of ERK, JNK, p38, and NF-κB by Western blotting for further study-specific effects of curcumin on the TLR4-MAPK/NF-κB signaling pathway.

## Results

### The characterization of curcumin-loaded CS-BSA NPs

**Fig. 1:**
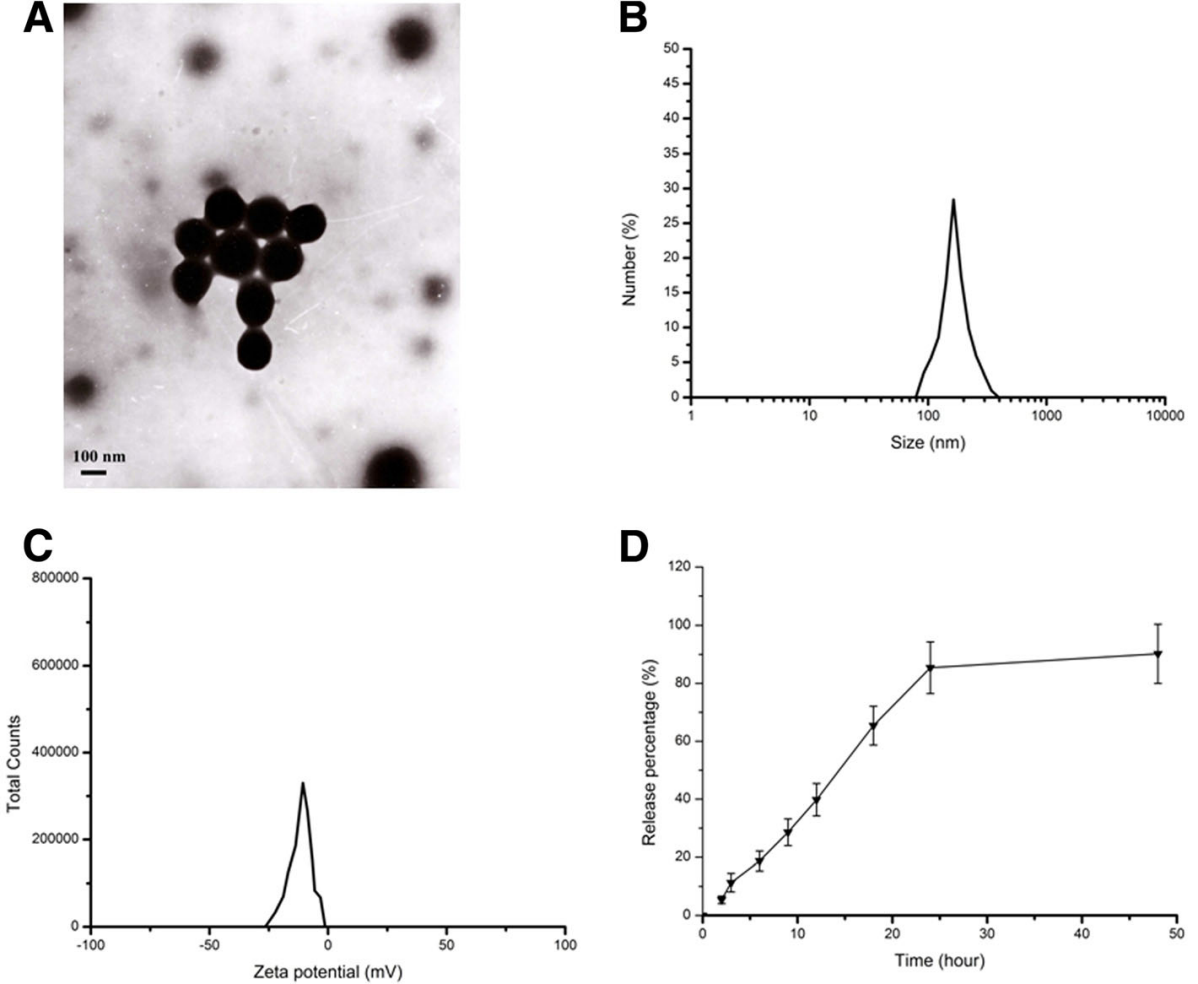
Characterization of curcumin loaded CS-BSA NPs. **a** TEM image of curcumin loaded CS-BSA NPs. **b** Dynamic light scattering (DLS) analysis of the obtained curcumin loaded CS-BSA NPs. **c** Zeta potential analysis of the obtained curcumin loaded CS-BSA NPs. **d** The in vitro release profile of the obtained curcumin-loaded CS-BSA NPs in phosphate-buffered saline with a pH of 7.4 at 37 °C for 48 h

The characteristics of NPs were investigated to determine their particle size, zeta potential, and morphology using a Zetasizer (Nano ZS90; Malvern Instruments, Malvern, UK) and transmission electron microscope (TEM) (Jeol, Tokyo, Japan) at an accelerating voltage of 200 kV. The results shown in Fig. 1 indicated that CS-BSA NPs presented a mean size at 143.5 nm, a negative zeta potential at − 10.8 mV, and a polydispersity at 0.021, respectively. It was observed that the curcumin-loaded CS-BSA NPs were spherical in shape and monodispersed. The resulting suspension containing curcumin-loaded CS-BSA NPs was centrifuged to obtain a supernatant solution to determine the absorbance of curcumin and to calculate the content of free curcumin in the supernatant solution according to the standard curve. The encapsulation efficiency (EE, %) of curcumin in NPs was valued at 95.4%. In terms of the drug-release process from the NPs, curcumin-loaded CS-BSA NPs showed a biphasic release pattern in the medium with a pH level of 7.4. About 11.3% of all drugs were released within the first 3 h, indicating that when the NPs entered the blood circulation ahead of reaching the BBB, curcumin was well protected and encapsulated in the core of the NPs. Furthermore, a few drugs were leaked from the NPs and released into the blood within the first 3 h. The majority of curcumin-loaded CS-BSA NPs could be transported around the BBB and enhanced the drug concentration around the brain.

### Penetration studies using an in vitro BBB model

**Fig. 2:**
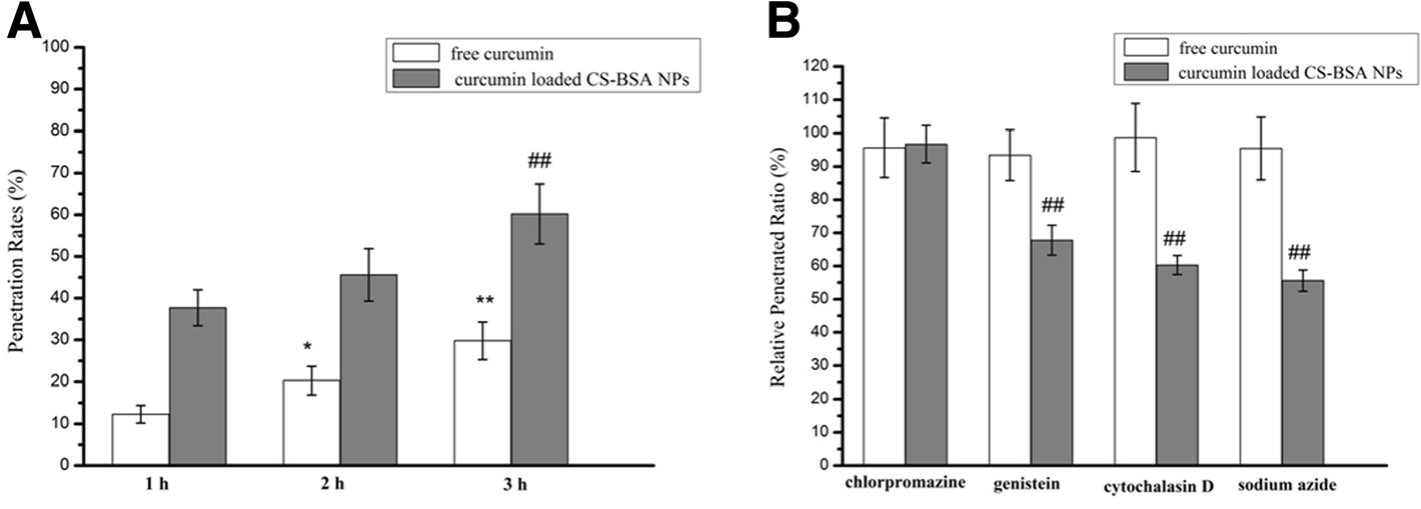
Analysis of the penetration mechanism of free curcumin and curcumin-loaded CS-BSA NPs through hCMEC/D3 cells. **a** Fluorescence spectrum analysis of the penetration rates of free curcumin and curcumin-loaded CS-BSA NPs. Results are expressed as means ± standard deviation (*n* = 3). *P < 0.05, **P < 0.01 vs the penetration rates of free curcumin at 1 h. ^##^*P* < 0.01 vs the penetration rates of curcumin loaded CS-BSA NPs at 1 h. **b** Effects of endocytic inhibitors on the penetrated ability of free curcumin and curcumin-loaded CS-BSA NPs. Results are expressed as means ± standard deviation (*n* = 3). ^##^*P* < 0.01 vs relative penetrated ratio of curcumin loaded CS-BSA NPs treated with chlorpromazine

The penetration rates of free curcumin and the NPs were assessed by checking the fluorescence intensity of curcumin in the lower chamber using a microplate reader (Synergy-2; BioTek Instruments), and they were calculated by determining the ratio of the fluorescence intensity of penetrated curcumin-loaded CS-BSA NPs in the lower chamber to that of the initially added curcumin-loaded CS-BSA NPs in the upper chamber. The results (Fig. 2) showed that the penetrating process of free curcumin and curcumin-loaded CS-BSA NPs followed time-dependent patterns, and the penetration rate increased with time. This suggested that the penetration rate of free curcumin were 12.3% at 1 h, 20.3% at 2 h, and 29.8% at 3 h. Compared with free curcumin, the penetrating efficiency of curcumin-loaded CS-BSA NPs was enhanced, as represented by the increased penetration rates; the penetration rates increased to 37.7% at 1 h, 45.6% at 2 h, and 60.2% at 3 h. This indicated that free curcumin may come across difficulties in penetrating through cells and showed poor permeability to the BBB [38, 39]. This observation also indicated that curcumin-loaded CS-BSA NPs could effectively promote drug penetration through cells, suggesting the role played by various endocytic pathways. An endocytosis inhibition test showed that being consistent with the previous reports [40], free curcumin depended on passive diffusion for penetration and that there was no obvious variation in the penetration efficiency of free curcumin, irrespective of whether an inhibitor was added or not. Conversely, the penetration of NPs was energy dependent, and the relative penetrated ratio treated with sodium azide was 55.6%. Furthermore, both caveolae and macropinocytosis primarily mediated the endocytic pathways of NPs. Compared with treatment with noninhibitors, the relative penetration ratios in cells treated with genistein and cytochalasin D were 67.8% and 60.3%, respectively.

### Cell apoptosis evaluation by MTT

**Fig. 3:**
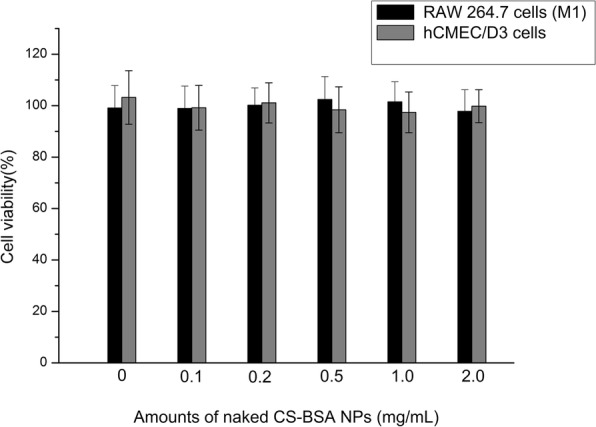
Viability of RAW 264.7 cells (M1) and hCMEC/D3 cells after incubation with different amounts of naked CS-BSA NPs for 24 h (*n* = 3)

The cytotoxic effects of blank CS-BSA NPs against RAW 264.7 cells (M1) and hCMEC/D3 were estimated in vitro by an MTT assay. Cells were treated with various concentrations of CS-BSA NPs that ranged from 0 to 2.0 mg/mL. A cell viability assay in Fig. 3 showed that no obvious cytotoxic activities were observed in RAW 264.7 cells and hCMEC/D3 cells when treated with blank CS-BSA NPs [37].

### Distribution and cellular uptake of NPs

**Fig. 4:**
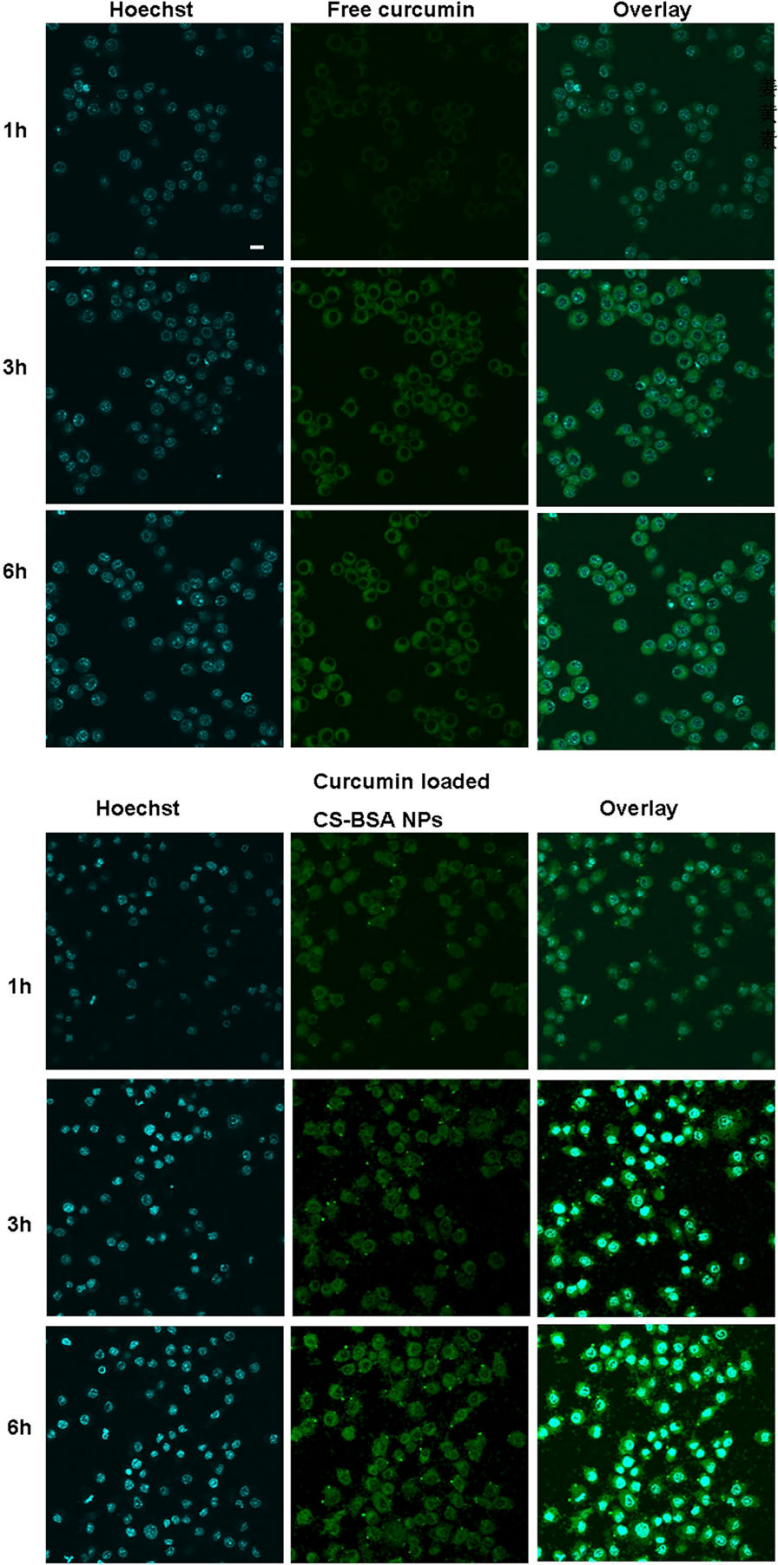
The uptake of free curcumin and curcumin loaded CS-BSA NPs in RAW 264.7 cells (M1) for 6 h. Curcumin showed green fluorescent color and indicated the intracellular location of free curcumin and curcumin loaded CS-BSA NPs. The nucleus was stained with Hoechst (blue) for 15 min at 37 °C. The scale bar is 50 μm and applies to all figure parts

Free curcumin and curcumin-loaded CS-BSA NPs containing the same amount of curcumin at 100 μg/mL were used to treat RAW 264.7 cells (M1), and the distribution and cellular uptake of curcumin were observed by confocal laser scanning microscopy (FluoView FV10i; Olympus). It can be seen in Fig. 4 that the intracellular distribution of free curcumin and curcumin-loaded CS-BSA NPs followed a time-dependent pattern and that the green fluorescence of curcumin had accumulated within the cell and dispersed throughout the entire cytoplasm. With the passage of time, the green fluorescence intensity inside the cells was enhanced. This demonstrated that the green fluorescence emitted by free curcumin inside the cells was very weak, which indicated that most of the free curcumin was not taken up by the macrophage cell line, RAW 264.7. As NPs might have promising potential for highly efficient intracellular drug delivery [41, 42], it was observed that curcumin-loaded CS-BSA NPs showed increased fluorescence intensity when compared with cells treated with free curcumin, suggesting that CS-BSA NPs could improve the cellular uptake of curcumin. Herein, this observation indicated that CS-BSA NPs could effectively promote drug accumulation within the cells.

### Phagocytosis of Aβ 42 induced by free curcumin and curcumin-loaded CS-BSA NPs

**Fig. 5:**
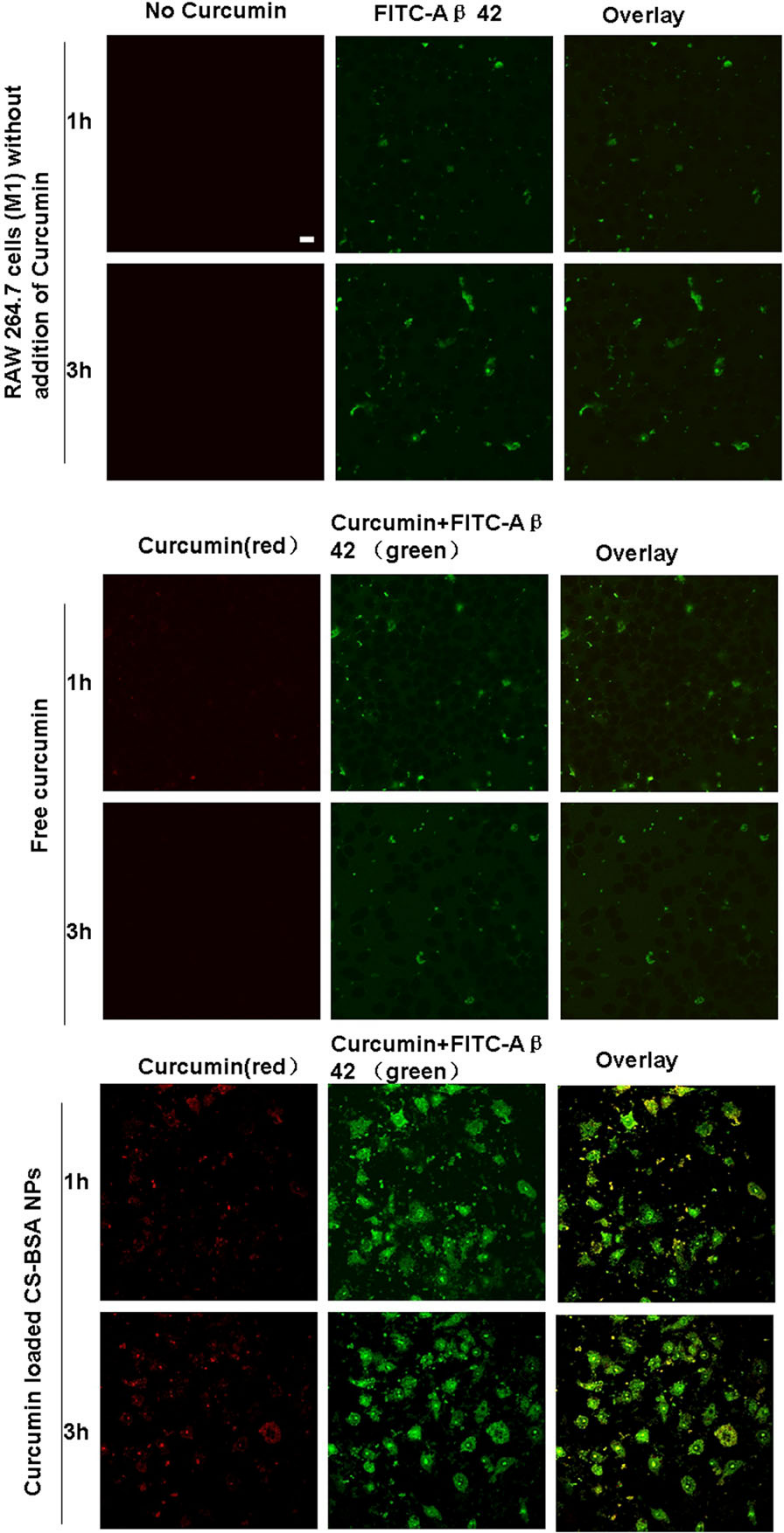
Phagocytosis of Aβ 42 induced by free curcumin and curcumin loaded CS-BSA NPs. The scale bar is 50 μm and applies to all figure parts

As shown in Fig. 5, curcumin displayed red fluorescence at an excitation wavelength of 550 nm and an emission wavelength of 570 nm. In addition, it also showed green fluorescence at the excitation and emission wavelengths of FITC. Therefore, once curcumin- and FITC-labeled Aβ 42 were phagocytized into RAW 264.7 cells (M1), they all showed green fluorescence at the excitation and emission wavelengths of FITC. In the co-localization experiment, red fluorescence and green fluorescence (representing curcumin) were merged and the yellow dots represented the intracellular existence and location of curcumin; some green dots were not co-located with the red fluorescent dots, representing the existence and phagocytosis of FITC-labeled Aβ 42 in RAW 264.7 cells (M1). It was observed that few amounts of Aβ 42 was phagocytized by microglia [43], which was proven by the intracellular observation of green fluorescence in the cells. As shown in the overlaying image in Fig. 5, compared with free curcumin, more of the yellow fluorescent dots of curcumin had been accumulated inside the cells, suggesting that a large amount of curcumin-loaded CS-BSA NPs, as represented by yellow fluorescent intensity, had been accumulated in the cells due to the interaction between NPs and RAW 264.7 cells (M1). This induced higher intracellular concentrations of curcumin, leading to the increased phagocytosis of Aβ 42 [44], as represented by the higher green fluorescent intensity. It was supposed that curcumin-loaded CS-BSA NPs induced macrophage polarization, as well as anti-inflammatory and neuroprotective effects contributed to the enhanced phagocytosis.

### Western blot assay

To explore the possible molecular mechanisms of curcumin-mediated macrophage polarization, we examined the overall levels of TNF-α, IL-6, and TLR4 as well as phosphorylation levels of p38, ERK, JNK, and IκBα by Western blotting to further study the specific effects of curcumin on the TLR4-MAPK/NF-κB signaling pathway.

**Fig. 6:**
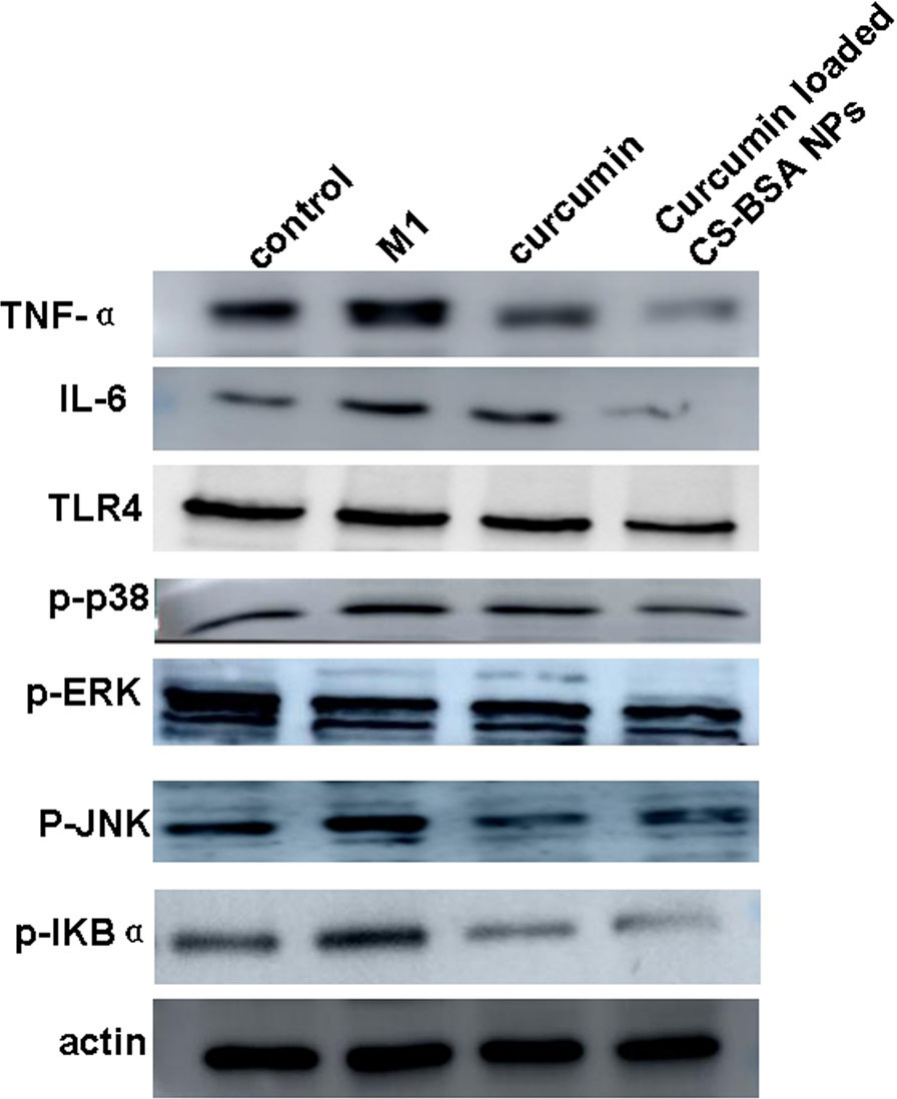
Western blot analyses of the expression levels of TNF-α, IL-6, TLR4, and phosphorylation of ERK, JNK, p38, and nuclear factor (NF)-κB in RAW 264.7 cells (M1 phenotype) after treatments with free curcumin and curcumin loaded CS-BSA NPs

Figure 6 showed that when compared with the normal RAW 264.7 cells as a control group, RAW 264.7 cells (M1 phenotype) released more potentially neurotoxic soluble factors and pro-inflammatory cytokines characterized by the higher expression levels of TNF-α and IL-6, thus leading to neuronal death and exacerbating the development of AD. Compared with control and free curcumin, curcumin-loaded CS-BSA NPs appeared to inhibit M1 macrophage polarization and induced the lowest expression of TNF-α and IL-6 in RAW 264.7 cells (M1 phenotype). Furthermore, curcumin-loaded CS-BSA NPs also decreased TLR4 expression, which regulated M1 macrophage polarization and the phosphorylation of ERK, JNK, p38, and NF-κB appeared to be reduced. This suggests that curcumin-loaded CS-BSA NPs effectively promoted curcumin accumulation—and its subsequent intracellular concentration—within the cells, thus enhancing its blocking effects on the TLR4-MAPK/NF-κB signaling pathway and further inhibiting M1 macrophage polarization.

## Discussion

AD is one of the most common neurodegenerative disorders and the main cause of death in developed countries. With respect to the treatment of AD, the BBB’s ability to prevent the entry of most exogenous substances in the brain was the main obstacle preventing their use. To address this problem, we designed CS-BSA NPs to improve the transportation of curcumin through the BBB. The results demonstrated that being consistent with the previous study [45], free curcumin difficultly penetrated through the cells and came to cross BBB, thus resulting in lower penetrating effects. Curcumin-loaded CS-BSA NPs could effectively promote drug penetration through the BBB with the mediation of caveolae- and micropinocytosis-mediated pathways. Neuroinflammation induced by Aβ aggregation is one of the critical factors underlying the pathological mechanisms of AD [46]. Aβ levels in the brain are determined by the dynamic balance between the generation and clearance of Aβ; therefore, the removal of Aβ is also important in determining its level in the brain. The phagocytosis ability of the microglia displayed important physiological significance in the prevention of AD. The microglia were mainly responsible for Aβ-target clearance and tended to predominantly aggregate around the deposition zone of Aβ 42, thus further preventing the accumulation of Aβ 42 by phagocytosis [47, 48]. The results showed that the phagocytosis effects of curcumin-loaded CS-BSA NPs-treated RAW 264.7 cells (M1 phenotype) on Aβ 42 was increased and that the accumulation and deposition of Aβ 42 were reduced, which may ameliorate the development of AD.

The microglia (M1 phenotype) releases potentially neurotoxic soluble factors and pro-inflammatory cytokines such as TNF-α and IL-6, thus leading to neuronal death and exacerbating the development of AD [49]. It is found that M1 macrophage polarization depends on the activation of the TLR4-MAPK /NF-κB signaling pathway and that blocking the TLR4-MAPK /NF-κB signaling pathway could inhibit M1 macrophage polarization and promote the polarization of macrophages from the M1 type to the M2 type [50]. Our results showed that curcumin as a component of the Indian spice, turmeric (Curcumin longa)—a type of ginger—was a potent anti-inflammatory agent that could reduce inflammation and may even play a role in AD treatment. CS-BSA NPs served as powerful tools for the efficient BBB-targeted penetration of curcumin. Compared with free curcumin, curcumin-loaded CS-BSA NPs induced phagocytosis effects and a larger amount of Aβ 42 was phagocytized by RAW 264.7 cells. In addition, curcumin-loaded CS-BSA NPs induced the lower protein expression levels of TNF-α, IL-6, and TLR4 than free curcumin, and the phosphorylation of ERK, JNK, p38, and NF-κB was also inhibited. It indicated that curcumin-loaded CS-BSA NPs may enhance curcumin-induced macrophage phagocytosis by inhibiting M1 macrophage polarizatione through blocking the TLR4-MAPK/NF-κB signaling pathway, thus promoting curcumin’s anti-inflammatory and neuroprotective effects.

## Conclusion

Our data suggested that curcumin could be used as a therapeutic agent in the treatment of AD. Curcumin-loaded CS-BSA NPs triggered the RAW 264.7 cell-induced phagocytosis of Aβ 42 by the enhanced BBB penetration of curcumin and higher intracellular drug concentration. In addition, curcumin-loaded CS-BSA NPs induced anti-inflammatory and neuroprotective effects by inhibiting M1 macrophage polarization and blocking the TLR4-MAPK/NF-κB signaling pathway. Taken together, curcumin-loaded CS-BSA NPs demonstrated their potential in enhancing the treatment of AD.

## Abbreviations

AD: Alzheimer’s disease
BBB: Blood–brain barrier
BSA: Bovine serum albumin
CS: Chitosan
LPS: Lipopolysaccharide
NPs: Nanoparticles
TLRs: Toll-like receptors
